# MiR218 Modulates Wnt Signaling in Mouse Cardiac Stem Cells by Promoting Proliferation and Inhibiting Differentiation through a Positive Feedback Loop

**DOI:** 10.1038/srep20968

**Published:** 2016-02-10

**Authors:** Yongshun Wang, Jingjin Liu, Jinjin Cui, Meng Sun, Wenjuan Du, Tao Chen, Xing Ming, Lulu Zhang, Jiangtian Tian, Ji Li, Li Yin, Fang Liu, Zhongyue Pu, Bo Lv, Jingbo Hou, Bo Yu

**Affiliations:** 1 Cardiology Department, Second Affiliated Hospital of Harbin Medical University, Harbin, Heilongjiang Province, China; 2 Key Laboratories of the Education Ministry for Myocardial Ischemia Mechanisms and Treatment, Harbin, Heilongjiang Province, China

## Abstract

MiRNA expression was determined in both proliferating and differentiated cardiac stem cells (CSCs) through a comprehensive miRNA microarray analysis. We selected miR218 for functional follow-up studies to examine its significance in CSCs. First, we observed that the expression of miR218 was altered in CSCs during differentiation into cardiomyocytes, and transfection of an miR218 mimic or miR218 inhibitor affected the myocardial differentiation of CSCs. Furthermore, we observed that a negative regulator of Wnt signaling, sFRP2, was a direct target of miR218, and the protein levels of sFRP2 were increased in cells transfected with the synthetic miR218 inhibitor. In contrast, transfection with the miR218 mimic decreased the expression of sFRP2 and potentiated Wnt signaling. The subsequent down-regulation of sFRP2 by shRNA potentiated Wnt signaling, contributing to a gene expression program that is important for CSC proliferation and cardiac differentiation. Specifically, canonical Wnt signaling induced miR218 transcription. Thus, miR218 and Wnt signaling were coupled through a feed-forward positive feedback loop, forming a biological regulatory circuit. Together, these results provide the first evidence that miR218 plays an important role in CSC proliferation and differentiation through the canonical Wnt signaling pathway.

Ischemic heart disease is one of the most prominent health problems worldwide and is associated with a high mortality rate. Stem cell therapy may directly regenerate cardiac tissue through the induction of neovasculogenesis and cardiogenesis. Cardiac stem cells (CSCs) are self-renewing, clonal proliferative stem cells and were first described in 2002 in the mouse heart^1^. CSCs have the potential for pluripotent differentiation into three major cardiac lineages: cardiomyocytes, endothelial cells, and vascular smooth muscle cells^2^,^3^,^4^. Resident CSCs may be particularly suitable for restoring dead myocardium because these cells are endogenous components of the adult heart, and they appear to be responsible for the physiological and pathological turnover of cardiac myocytes and other cardiac cells^5^. In particular, CSCs expressing the stem cell factor (SCF) receptor c-kit have been extensively characterized and are effective in intracoronary transplantation, improving myocardial function^6^. Previous studies have shown that human CSCs predominantly differentiate into cardiomyocytes and, to a lesser extent, into smooth muscle cells and endothelial cells in the infarcted hearts of rats^7^. Clinical studies have also demonstrated the safety and secondary efficacy of human CSCs in patients with heart failure^8^.

Although resident CSCs might represent a logical source for cardiomyocyte differentiation, the efficacy of CSCs in myocardial regeneration after MI remains uncertain, partly due to the extremely low abundance of these cells^9^. Moreover, the efficiency of CSCs is often hampered by a lack of successful myocardium differentiation. The effective transdifferentiation of stem cells is likely to be highly dependent on regulating growth factors via the administration of exogenous factors or molecular programming of stem cells. For example, Wnt proteins are growth factors that function during embryonic development and in adults through the regulation of diverse cellular processes, such as gene transcription and cell proliferation, migration, polarity, and division^10^,^11^. Not surprisingly, Wnt proteins are also involved in cardiac development and differentiation. The positive involvement of Wnt/β-catenin signaling in cardiogenesis has been demonstrated in Drosophila^12^. In addition, cell culture-based experiments have suggested a positive role for canonical Wnt in early cardiac differentiation^13^. In contrast, inhibition of canonical Wnt signaling at this early time point leads to inhibition of cardiac differentiation and a reduction of contractile areas within embryoid bodies at later time points^14^. These findings suggest a model in which Wnt/β-catenin signaling has a biphasic function during cardiogenesis^14^,^15^,^16^. Furthermore, cell autonomous and non-autonomous effects should be considered, because much of the relevant data has come from experiments performed in ES cell cultures harboring different but communicating cell types or from non-tissue-specific gain or loss-of-function experiments in frogs or chickens. In the present study, the isolation and analysis of progenitor cell populations defined according to specific markers or the tissue-specific modulation of Wnt signaling were taken as the gold standard for further investigations.

Micro-RNAs (miRNAs) are small non-coding RNAs that inhibit translation or promote mRNA degradation through binding to the 3′ untranslated region (3′ UTR) of target mRNAs, resulting in “fine-tuning” of gene expression^17^,^18^. MiRNAs display additive effects and reduce the dependence on a single molecular target to elicit a functional response. Thus, the transcriptional regulation of host genes results in posttranscriptional regulation of a set of target genes that enhance or diminish host gene activity. Previous studies have shown that miRNAs play a critical role in cardiogenesis and cardiac regeneration and regulate the canonical Wnt pathway^19^,^20^. Several studies have focused on the differential expression of miRNAs in cardiac pathologies, identifying clusters of differentially expressed miRNAs among various human cardiomyopathies^21^. Furthermore, the contribution of specific miRNAs to CSC differentiation has previously been described^22^,^23^,^24^. Accumulating evidence indicates that miRNAs are necessary, are easily controlled and efficiently target CSC proliferation and differentiation.

In the present study, we demonstrated that miR218 is necessary for CSC proliferation and cardiac differentiation, and that expression of the members of the miR218 family increases during proliferation and cardiac differentiation. Moreover, secreted frizzled-related protein 2 (sFRP2), the negative regulator of Wnt signaling, was a direct target of miR218. Therefore, to verify the impact of miR218 on cardiogenesis, we selected CSCs as a more advanced model compared with other cell lines. The results showed that miR218 and Wnt signaling promotes CSC proliferation and suppresses differentiation. Furthermore, we observed that miR218 activates Wnt signaling through a reduction of sFRP2 expression.

## Results

### Expression of miRNAs in proliferating and differentiated CSCs

**Figure 1:**
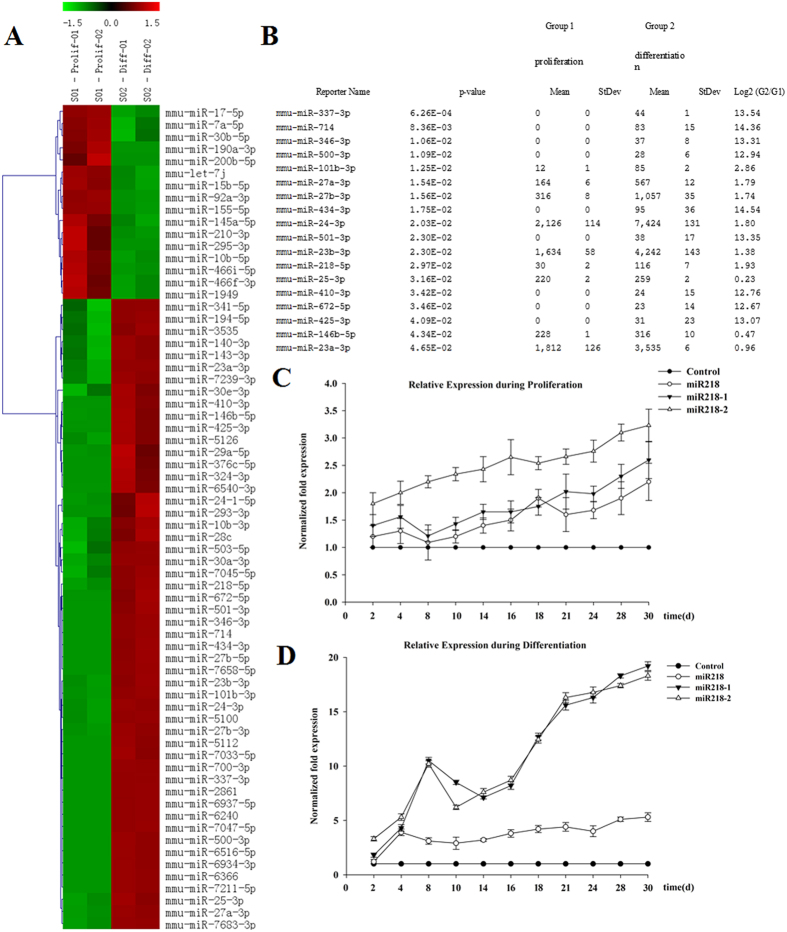
Expression of miRNAs in proliferating and differentiated CSCs. (**A**) miRNA heat map of proliferating CSCs and CSCs that have differentiated into cardiomyocytes. The color scale illustrates the relative expression level of an miR across all samples: red represents expression level higher than the mean, and green represents expression lower than the mean. The analysis was performed on the log2 (G2/G1) signal intensity ratios (where G1 represents proliferating cells and G2 represents differentiated cells) passing the filtering criteria on variation across samples; P-value < 0.1. (**B**) miRNAs showing statistically significant differences in expression in differentiated CSCs. (**C**,**D**) Total RNA was analyzed for the expression of miR218 and its precursors miR218-1 and miR218-2 through real-time PCR at the indicated time points during proliferation (**C**) or differentiation (**D**). Expression was normalized to the U6 small RNA.

MiRNA expression was determined in both proliferating and differentiated CSCs through comprehensive miR microarray analysis. Sixty-eight miRNAs out of the ~1000 miRNA molecules on the chip were detected, among which 16 miRNAs were detectable in proliferating CSCs, and 52 miRNAs were detectable in differentiated CSCs (Fig. 1A). A total of 18 miRNAs were significantly differentially expressed in differentiated CSCs (Fig. 1B). Among these miRNAs, the expression of 10 miRNAs (miR337, miR714, miR346, miR500, miR101b, miR434, miR501, miR410, miR672 and miR425) was significantly decreased, and we therefore did not further examine the expression levels of these molecules by using quantitative RT-PCR (qRT-PCR). The expression of the remaining 8 miRNAs was assessed via qRT-PCR, revealing that the expression of miR218 was relatively higher in differentiating cells. Notably, the role of miR218 in the differentiation of CSCs has not yet been studied. qRT-PCR analysis was performed to confirm the level of miR218 during cell proliferation and differentiation, and surprisingly, the levels of miR218 were shown to be upregulated (Fig. 1C,D), as were the levels of the pre-miRNAs miR218-1 and 218-2. Therefore, we selected miR218 for functional follow-up studies to examine the significance of this molecule for proliferation and differentiation in CSCs.

### MiR218 is involved in the regulation of cell proliferation and differentiation

**Figure 2:**
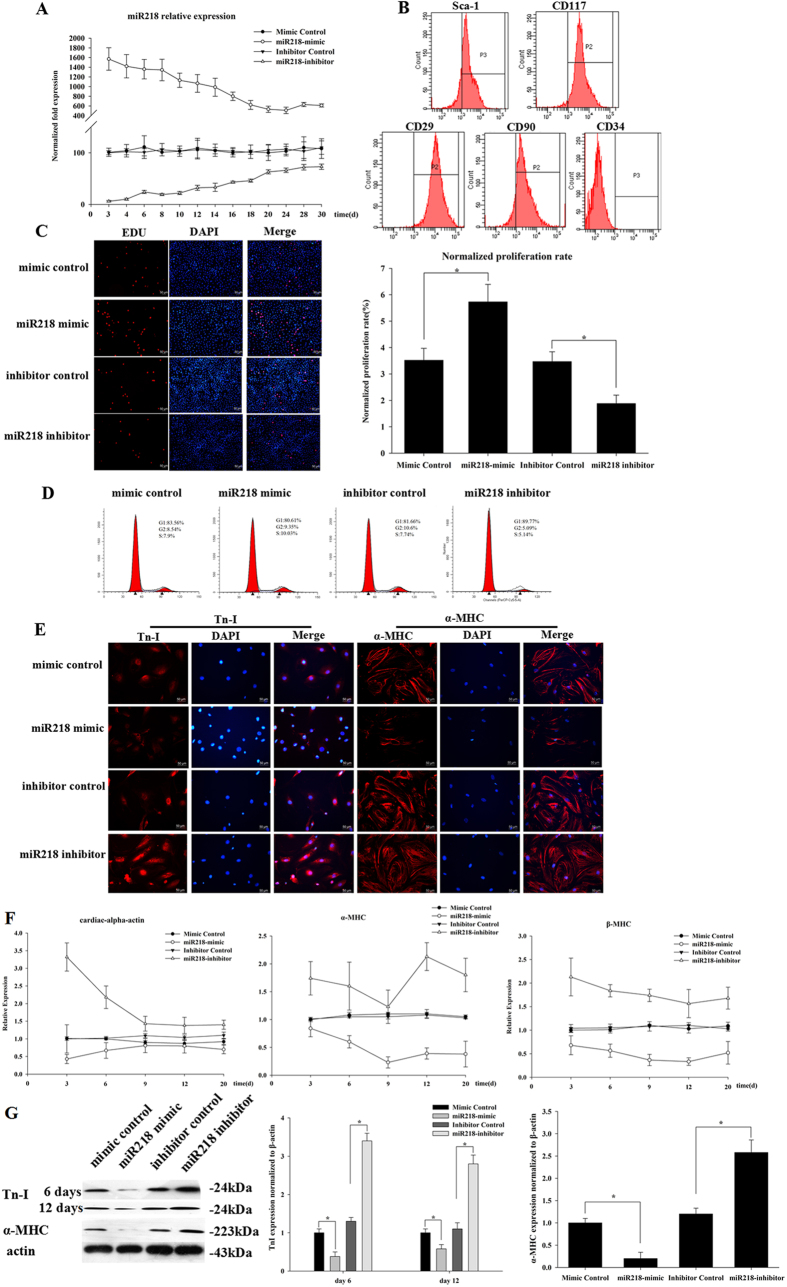
MiR218 promotes cell proliferation and inhibits cardiac differentiation. (**A**) Transfection efficiency of miR218 was evaluated through qRT-PCR. Expression was normalized to U6. (**B**) Characterization of surface markers on CSCs identified through flow cytometry after 30 passages in culture. The results are shown as the means ± SD of at least three separate experiments. (**C**) The cells were stained with EdU and Hoechst 33342 after transfection with the miR218 mimic and miR218 inhibitor. (**D**) Cell cycle distribution of CSCs after transfection with the miR218 mimic and miR218 inhibitor for 48 h. The results are shown as the means ± SD of at least three separate experiments. (**E**) The effects of the miR218 mimic and the miR218 inhibitor were observed through immunofluorescence assays on days 6 (TnI) and 12 (α-MHC) of CSC differentiation. (**F**) CSCs were transfected with the miR218 mimic and the miR218 inhibitor. Total RNA was analyzed through qRT-PCR to determine the mRNA expression profile of myocardium marker genes at days 3, 6, 9, 12 and 20. Cardiac-α-actin, α-MHC and β-MHC were used as markers of early and late stages of myocardium development. The results are shown as the means ± SD of at least three separate experiments. (**G**) Western blot analysis of proteins from CSCs transfected with the miR218 mimic or the miR218 inhibitor and subsequently cultured in differentiation media for 12 days. The protein profiles were normalized to β-actin.

In the present study, we sought to examine whether miR218 affects cell proliferation and cardiac differentiation in CSCs. The functional activity of miR218 was assessed in CSCs after forced expression of an miR218 mimic and an miR218 inhibitor. After transfection, miR218 expression levels were examined through qRT-PCR, which demonstrated that miR218 expression continued for 30 days (Fig. 2A). To ensure that all of the proliferating cells were cardiac stem cells, the cells were identified via flow cytometry. The results showed that 96.76 ± 1.6% of cells were c-kit + (Fig. 2B), but these cells were negative for CD34 (0.2 ± 0.12%). In addition, these cells were positive for the control mesenchymal markers CD90 (89.66 ± 3.2%) and CD29 (99.43 ± 0.2%) and the stemness marker sca-1 (90.88 ± 2.07%) (Fig. 2B), as expected. We next evaluated the proliferation of CSCs by using EdU and flow cytometry. The EdU results showed that the miR218 mimic group was more proliferative (5.73% ± 0.66%) than the control group (3.52% ± 0.45%), whereas the inhibitor group (1.88% ± 0.32%) exhibited lower proliferation compared with the control group (Fig. 2C). We next examined the DNA context by using flow cytometry in miR218 mimic- and inhibitor-treated CSCs. We observed that transfection of the miR218 mimic promoted cell proliferation to a greater extent (10.5 ± 0.5%, S phase), as compared with the control group (7.8% ± 0.36%), whereas silencing miR218 decreased cell proliferation (5.08 ± 0.34%, S phase) as compared with the control group (7.51 ± 0.33%, S phase) (Fig. 2D). The data also showed that the miR218 mimic promoted G1 to S transition in CSCs.

The differentiation of CSCs is characterized by two stages of phenotypic maturation, corresponding to day 6 (early differentiation) and day 12 (late differentiation). Overexpression of the miR218 mimic decreased differentiation, whereas overexpression of the inhibitor increased differentiation, as reflected by TnI and α-MHC, two markers of myocardium maturation (Fig. 2E). Immunofluorescence results showed that the expression of TnI and α-MHC was decreased in the miR218 mimic group and elevated in the inhibitor group. However, the expression of α-MHC was not detected on day 6. Similarly, the mRNA expression of α-MHC, β-MHC, and cardiac-α-actin was significantly decreased on days 3, 6, 9, 12 and 20 in the miR218 mimic group but was increased in the inhibitor group (Fig. 2F). The protein expression of TnI and α-MHC was detected through western blot analysis at days 6 and 12. Compared with that of the control group, the expression of TnI and α-MHC was reduced 0.5-fold in the miR218 mimic group and increased approximately 2-fold in the miR218 inhibitor group (Fig. 2G).

Therefore, these data showed that miR218 promotes cell proliferation and inhibits the cardiac differentiation of CSCs.

### SFRP2 is a target of miR218

**Figure 3:**
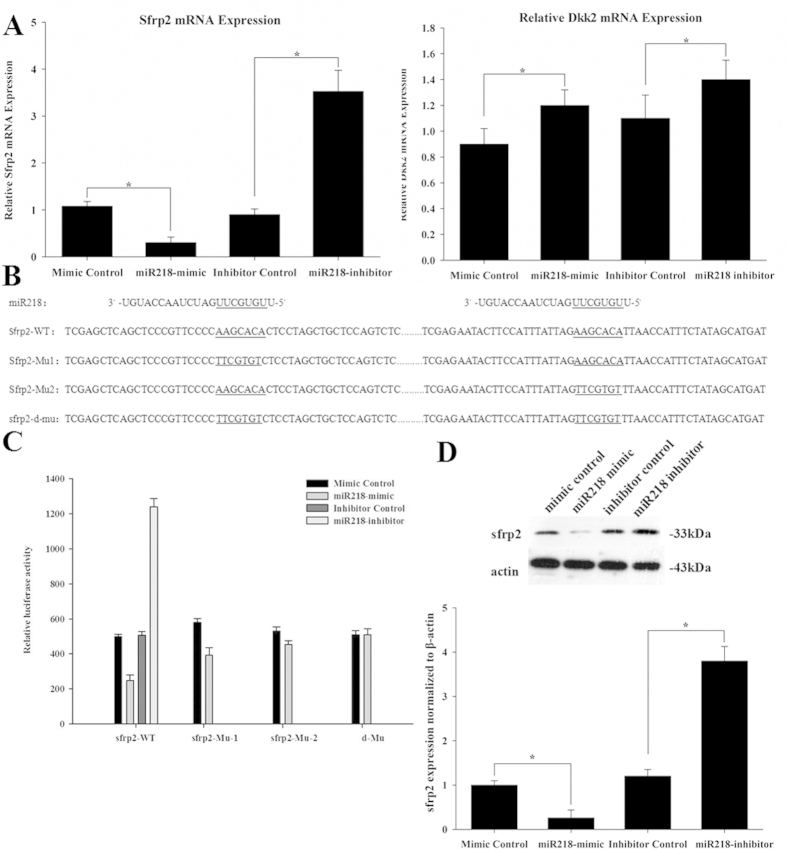
SFRP2 is a validated target of miR218 in CSCs. (**A**) Regulation of endogenous expression of sFRP2 and Dkk2 by the miR218 mimic and miR218 inhibitor. Analysis of total RNA to determine the relative expression of miR218 targets through qRT-PCR in CSCs at 48 h after transfection using the mimic-control, miR218 mimic, inhibitor-control and miR218 inhibitor. Relative expression levels were normalized to GAPDH levels. (**B**) The nucleotide sequence of miR218, the predicted binding sites of miR218 and the mutated nucleotides (underlined) in the 3′ UTR of sFRP2. (**C**) Dual luciferase activity in transfected CSCs. (**D**) Western blot analysis of proteins from CSCs transfected with the miR218 mimic and the miR218 inhibitor. The protein profiles were normalized to β-actin.

To address the mechanism through which miR218 regulates cardiac differentiation, we examined the predicted targets of miR218. The putative target sites of miR218 were predicted by using TargetScan, microRNA and PicTar software. Among all of the possible targets, several potential targets meeting this criterion were determined to be top putative targets. From these genes, we selected two Wnt inhibitors, Dkk2 and sFRP2, for further study, because these proteins are potentially involved in proliferation and differentiation through the Wnt signaling pathways. We therefore examined the mRNA expression of each target in response to the miR218 mimic and the miR218 inhibitor. We observed that the mRNA expression of sFRP2, but not Dkk2, was significantly changed (Fig. 3A). Therefore, sFRP2 was selected as the better candidate between the two proteins. There are two possible binding sites in the sFRP2 3′ UTR. To determine whether miR218 directly regulates sFRP2, we performed dual luciferase reporter experiments and observed that the luciferase activity of sFRP2-WT was markedly reduced after transfection with the miR218 mimic for 24 h. However, single and double mutations partially or completely abolished the repression induced by miR218 (Fig. 3C), indicating that miR218 could specifically target the binding sites in the 3′ UTR of sFRP2. The miR218 binding site in the seed sequences within the 3′ UTR of sFRP2 mRNA is illustrated in Fig. 3B. We also examined the protein expression levels of sFRP2 in response to miR218. We observed that sFRP2 protein levels were significantly decreased by treatment with the miR218 mimic and increased by treatment with the miR218 inhibitor (Fig. 3D). Together, these results indicated that sFRP2 is a direct target of miR218 in CSCs.

### MiR218 regulates cell proliferation and differentiation through targeting sFRP2

**Figure 4:**
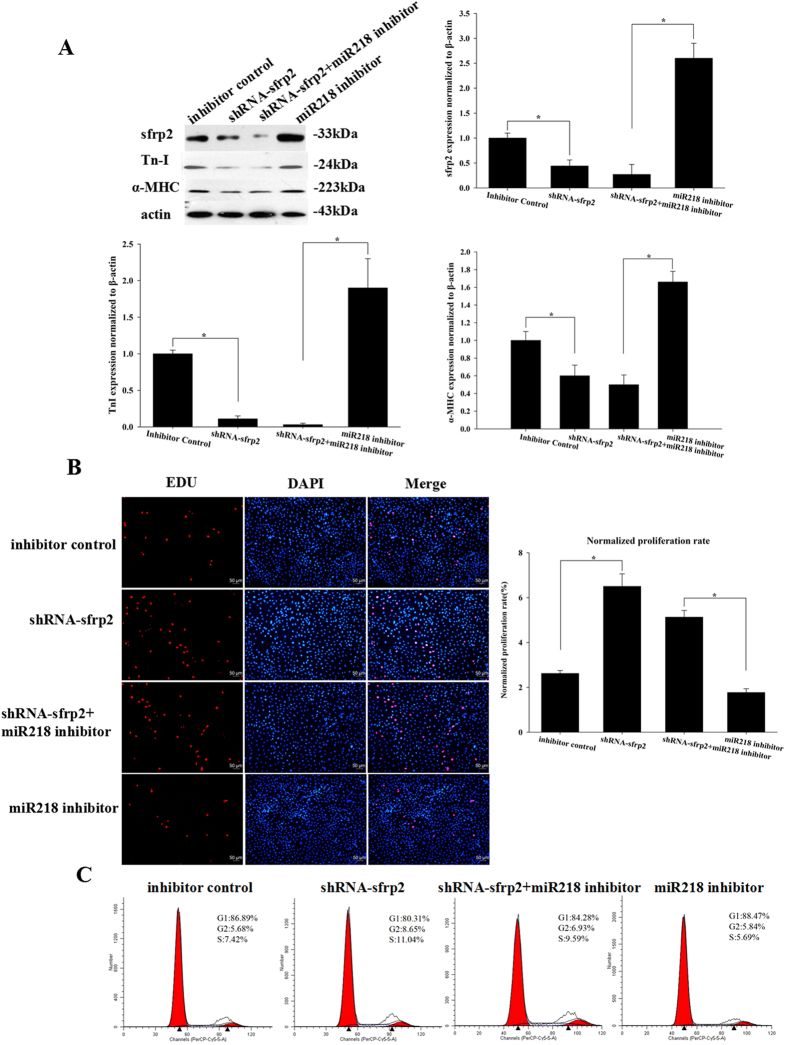
MiR218 regulates cell proliferation and differentiation by targeting sFRP2. (**A**) Western blot analysis of proteins from CSCs transfected with sFRP2 shRNA, the miR218 inhibitor+sFRP2 shRNA and the miR218 inhibitor and subsequently cultured in differentiation media for 12 days. The protein profiles were normalized to β-actin. (**B**) The cells were stained with EdU and Hoechst 33342. (**C**) Cell cycle distribution of CSCs after transfection with sFRP2 shRNA, the miR218 inhibitor+sFRP2 shRNA and the miR218 inhibitor for 48 h.

To determine whether the regulation of cell proliferation and differentiation through miR218 is directly mediated by sFRP2, we transfected the miR218 inhibitor and sFRP2 shRNA into CSCs and examined sFRP2 expression levels through western blotting (Fig. 4A). EDU and cell cycle assays demonstrated that the sFRP2-shRNA group was more proliferative (6.5% ± 0.56% and 11.04 ± 0.5%, respectively) than the inhibitor control group (2.62% ± 0.13% and 7.43 ± 0.42%, respectively). The proliferation results also suggested that cells transfected with the combination of the miR218 inhibitor and sFRP2 shRNA (5.13% ± 0.30% and 9.59% ± 0.67%, respectively) showed higher proliferation than those treated with the miR218 inhibitor (1.77% ± 0.17% and 5.69 ± 0.33%, respectively) (Fig. 4B), whereas differentiation was restricted in the combined transfection group compared with miR218 inhibitor treatment. These results suggested that sFRP2 knockdown following miR218 inhibition reduced cell differentiation and promoted cell proliferation. These data confirmed that the effects of miR218 on cell proliferation and differentiation could be attributed to the target protein sFRP2.

### MiR218 is a positive regulator of canonical Wnt signaling

**Figure 5:**
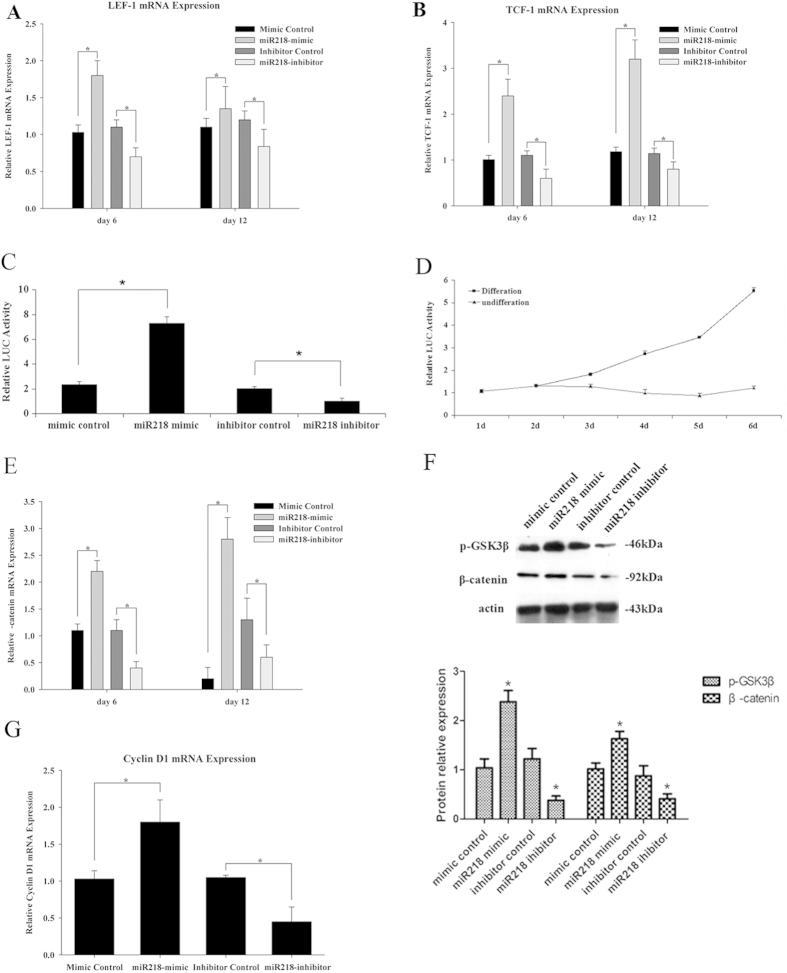
MiR218 is a positive regulator of canonical Wnt signaling. (**A**) Expression of Tcf-1 mRNA, (**B**) Lef-1 mRNA, (**E**) β-catenin mRNA and (**G**) CyclinD1 mRNA in CSCs transfected with the miR218 mimic and the niR218 inhibitor. The cells were analyzed on the indicated days. Relative expression levels were normalized to GADPH levels. (**C**) The miR218 mimic and miR218 inhibitor affect Wnt reporter activity. The WT Wnt TOPflash reporter was transfected into CSCs together with the mimic-control, miR218 mimic, inhibitor-control and miR218 inhibitor for 24 h. Relative luciferase activity was measured and plotted. (**D**) Wnt signaling activity in these cells during differentiation, examined by using Super Top flash. (**F**) Western blot analysis of the proteins from CSCs transfected with the miR218 mimic and the miR218 inhibitor and subsequently cultured in differentiation media for 12 days. The protein profiles were normalized to β-actin.

As a blocker of canonical Wnt signaling, sFRP2 has various biological effects associated with the regulation of the Wnt signaling pathway. To identify the mechanisms associated with miR218 and Wnt signaling in cardiomyogenesis, we examined whether two transcriptional mediators of activated Wnt signaling, Tcf-1 and Lef-1, responded to miR218 (Fig. 5A,B). Quantitative RT-PCR results showed that treatment with the miR218 mimic increased endogenous Tcf-1 and Lef-1 expression, whereas treatment with the miR218 inhibitor decreased Tcf-1 and Lef-1 gene expression. A Wnt-responsive TOPflash reporter assay was performed to confirm that Wnt signaling was involved in the effects of miR218 on CSC proliferation and differentiation. The results showed that Wnt signaling activity was increased 3.1-fold in the miR218 mimic group and decreased 18% in the miR218 inhibitor group (Fig. 5C). Using the same assay method, we also observed that Wnt signaling activity increased greatly over time (Fig. 5D). We subsequently detected β-catenin mRNA expression levels in response to transfection with the miR218 mimic and miR218 inhibitor (Fig. 5E). Western blot analysis revealed that the levels of β-catenin and the phosphorylated GSk3β protein in the Wnt signaling pathway were significantly increased by the miR218 mimic and decreased by the miR218 inhibitor during CSC differentiation (Fig. 5F). In addition to the direct target of Wnt signaling, we examined another Wnt signaling target, Cyclin D1. Cyclin D1 was increased by the miR218 mimic and decreased by the inhibitor (Fig. 5G). Hence, the observed effect on the proliferation of miR218 might be associated with the Wnt signaling target Cyclin D1.

**Figure 6:**
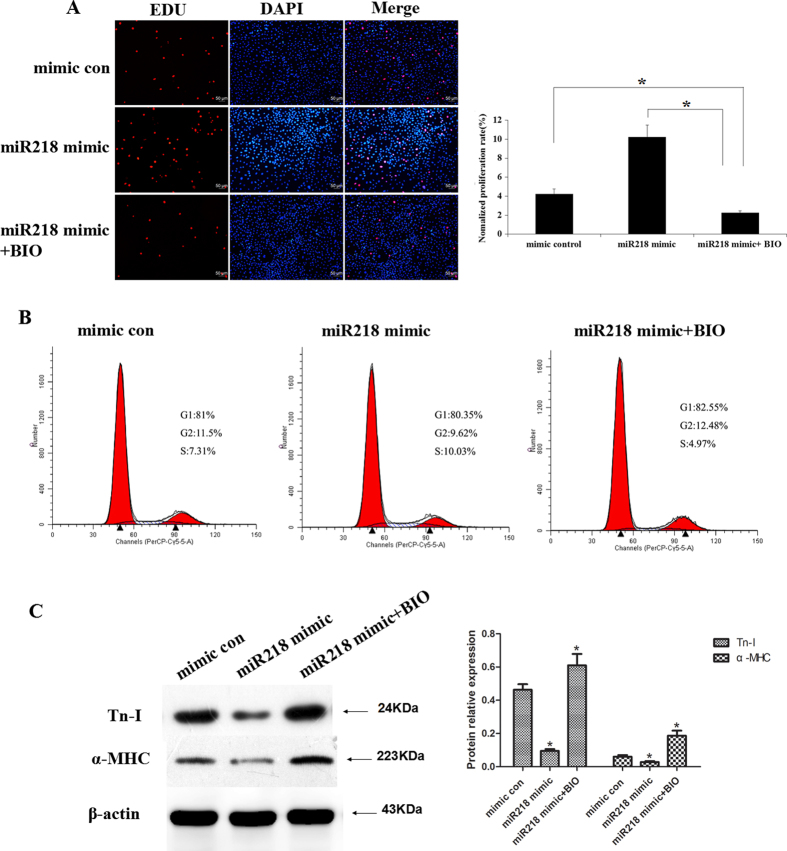
BIO restored the effects of miR218 on the proliferation and cardiac differentiation of CSCs. (**A**) Cells transfected with the miR218 mimic and treated with BIO were stained with EdU and Hoechst 33342. (**B**) Cell cycle distribution of the CSCs after the same treatment. (**C**) Western blot analysis of proteins from CSCs transfected with the mimic-control or the miR218 mimic or treated with the miR218 mimic and BIO and subsequently cultured in differentiation medium for 12 days.

To further confirm the effects of miR218 on CSCs through canonical Wnt signaling, we examined the proliferation and differentiation of CSCs after transfection with the miR218 mimic and treatment with 5 μM 6-Bromoindirubin-3′-oxime (BIO) (a Gsk3 inhibitor). EDU assays demonstrated that proliferation was significantly lower in the miR218 mimic and BIO treatment group (2.25 ± 0.32%) compared with the miR218 mimic-only group (10.25 ± 1.37%) or the mimic-control group (4.22 ± 0.42%) (p < 0.001, n = 3; Fig. 6A). Cell cycle assays showed consistent results (4.87 ± 0.32%, 10.66 ± 0.83% and 7.46 ± 0.54% S phase in the mir218 mimic and BIO treatment group, the mir218 mimic-only group and the mimic-control group, respectively, p < 0.001, n = 3; Fig. 6B). Similarly, BIO restored the differentiation of CSCs that was inhibited by the miR218 mimic (Fig. 6C).

### MiR218 modulated Wnt signaling in cardiomyogenesis through a positive feedback loop

**Figure 7:**
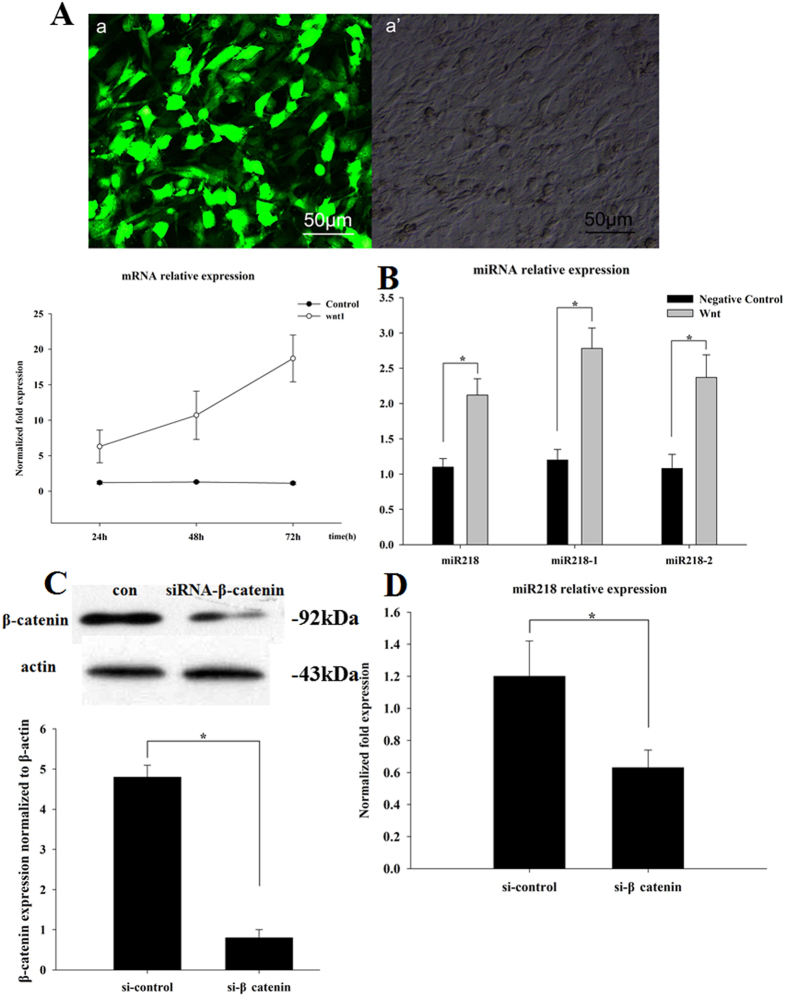
MiR218 modulates Wnt signaling in cardiomyogenesis through a positive feedback loop. (**A**) Fluorescence images (a) and microscopy images (a’) obtained after Wnt1 lentivirus infection. The expression of Wnt1 was detected through qRT-PCR. (**B**) The expression of miR218, miR218-1 and miR218-2 was detected through qRT-PCR after Wnt1 lentivirus infection, and all of the miR218 family members were found to be upregulated in response to the exogenous expression of Wnt1, the mediator of canonical Wnt signaling. Expression was normalized to U6. (**C**) Western blot analysis of proteins from CSCs transfected with siRNA-β-catenin for 48 h. Protein profiles were normalized to β-actin. (**D**) The expression of miR218 was detected through qRT-PCR with siRNA-β-catenin for 48 h. Expression was normalized to U6.

To investigate the functional roles of Wnt1 during cardiomyogenesis in CSCs, a Wnt1 gene expression lentivirus was constructed and used to infect CSCs. After transfection, Wnt1 expression levels were examined through fluorescence microscopy and qRT-PCR (Fig. 7A). Indeed, overexpression of the Wnt signaling pathway increased miR218, miR218-1 and miR218-2 levels by approximately 2.2-, 2.8- and 2.4-fold, respectively (Fig. 7B). We subsequently transfected CSCs with a β-catenin siRNA. Western blotting revealed that siRNA transfection decreased the β-catenin protein level 23-fold compared with that in siRNA-NC-treated cells (Fig. 7C). After transfection, knockdown of β-catenin decreased miR218 expression (Fig. 7D). These assays demonstrated that Wnt signaling induced miR218, and in turn, miR218 increased Wnt activity. Thus, miR218 and Wnt signaling are coupled through a forward-positive feedback loop and together form a biological regulatory circuit.

## Discussion

**Figure 8:**
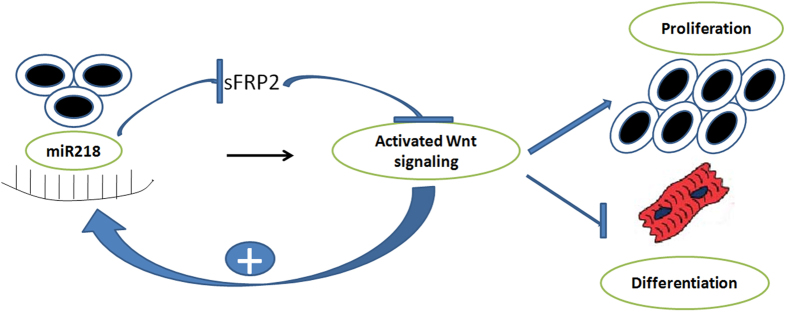
Proposed model. Illustration of the positive feedback loop between miR218 and Wnt signaling that constantly renews Wnt signaling through regulation of the Wnt inhibitor sFRP2. mir218 and canonical Wnt signaling regulate CSC proliferation and differentiation through a variety of mechanisms. Canonical Wnt signaling induces miR218 transcription. miR218 subsequently downregulates the key Wnt signaling antagonist sFRP2, thereby potentiating Wnt signaling. These two actions promote a gene expression program that is necessary for CSC proliferation and differentiation.

In the present study, we observed that CSCs expressed many different miRNAs, and some of these miRNAs were highly expressed after differentiation. Here, we focused on miR218 to determine whether this molecule could be used *in vitro* to modulate cardiomyocyte differentiation and proliferation. Reintroduction of miR218 significantly promoted the proliferation and inhibited the differentiation of CSCs. We established that miR218 acted as a central regulatory node that optimized Wnt signaling, thus regulating myocardial proliferation and differentiation. Here, we provided evidence of a novel positive feedback loop involving canonical Wnt signaling and miR218 that likely contributes to CSC differentiation and proliferation (Fig. 8). Therefore, the miR218/Wnt-signaling axis defines a molecular network that promotes myocardial proliferation and inhibits differentiation.

MiR218 has been frequently mentioned in association with human malignancies. For example, in acute and chronic lymphocytic leukemia, miR218 and miR-34a are considered to function as tumor suppressor miRNAs^25^,^26^. In recent years, miR218 has been implicated in the “fine-tuning” of Slit-Robo pathway genes and the generation of negative feedback in response to Slit gene activation^27^. Moreover, Fish *et al*.^28^ have indicated that miR218 and multiple Slit/Robo signaling components are required for heart tube formation in zebrafish, and this network modulates the previously unappreciated function of VEGF signaling during this process. Hassan *et al*.^29^ have observed that miR-218 promotes the differentiation of osteoblasts and osteomimicry of metastatic cancer cells through a Wnt signaling circuit. Zhang *et al*.^30^ have also shown that miR-218 and Wnt/β-catenin signaling promotes osteogenic differentiation of human adipose tissue-derived stem cells. The data obtained in the present study suggested that miR218 expression is negatively correlated with cardiomyocyte differentiation. Interestingly, the increased miR218 expression in the differentiating cells did not promote the differentiation of CSCs. Instead, as miR218 levels increased in differentiating cells, the differentiation capacity of the CSCs gradually disappeared. Therefore, miR218 might be used as a biomarker to evaluate CSC differentiation. In addition, many miRNAs that influence myocardial differentiation through the regulation of diverse signaling molecules and pathways remain undefined. For example, miR-1 is the most abundant miRNA in cardiomyocytes and has been identified as the first miRNA involved in heart development^31^. MiR-1 and miR-133, isolated from a common primary miRNA, are transcriptionally regulated through SRF and MEF2 and promote the expression of muscle-specific genes^22^,^32^,^33^. Another miRNA with an important function in heart development is miR-499, which represses the miR-499 target genes Sox6 and Rod1 in human CSCs, enhancing cardiomyogenesis *in vitro* and after infarction *in vivo*^24^. Interestingly, in embryonic stem cells, miR-499 upregulates the expression of heart-specific genes such as Nkx2.5 and MEF2C and regulates genes involved in the Wnt pathway^34^. Recently, miRNAs have been shown to regulate cardiomyocyte progenitor cell fate through a novel candidate, miR-204, whose levels are markedly increased during cardiomyocyte progenitor cell differentiation. Inhibition of miR-204 increases cardiomyocyte progenitor cell proliferation without affecting cell viability, reducing the differentiation of cardiomyocyte progenitor cells^35^. These studies highlight the wide range of signaling pathways modulated through miRNAs in cardiomyogenesis. Similarly, we demonstrated that miR218 promoted proliferation, but the precise impact on proliferation requires further investigation.

The functions of Wnt/β-catenin signaling during cardiac development in vertebrates are contradictory. In Xenopus, IGFBP-4 binds to sFRP2 and LRP6, thereby preventing the binding of a Wnt ligand. Interestingly, this inhibition of Wnt/β-catenin signaling is required for terminal differentiation^36^. However, a study examining cardiomyocytes has suggested that sFRP2 inhibits differentiation through inhibition of the transcriptional activation of Wnt3a^37^. This apparent conflict in the diverse roles of Wnt/β-catenin signaling was resolved with the observation that Wnt signaling has different effects depending on the time of action. For example, a study in heat shock-inducible transgenic zebrafish embryos has indicated that the application of Wnt8 before gastrulation increases the number of cardiomyocytes, whereas the application of Wnt8 after gastrulation reduces the number of cardiomyocytes. Previous publications have confirmed these observations in mouse ES cells. In this system, the expression of canonical Wnt ligands occurs slightly earlier than the expression of cardiac genes^13^,^14^. The specification of these CSCs is initiated at the onset of gastrulation in the mesodermal germ layer. Therefore, we proposed that the Wnt signaling pathway inhibits CSC differentiation and confirmed this hypothesis in the present study.

We observed that miR218 was upregulated in proliferating and differentiating CSCs, but it promoted the proliferation and inhibited the differentiation of CSCs. This effect might be the result of canonical Wnt signaling activation. During the differentiation experiment, we used special culture medium to induce CSC differentiation, and we observed that canonical Wnt signaling was strongly activated during differentiation. Furthermore, in subsequent experiments, we found that the activation of canonical Wnt signaling paralleled the increasing expression of miR218, whereas when the cells were transfected with the miR218 mimic, differentiation was inhibited. These results suggested that miR218 inhibited differentiation and that the Wnt signaling pathway may have been activated, thus leading to increased miR218 expression. However, the increase in miR218 expression was not sufficient to inhibit the differentiation of CSCs; additional miR218 expression was required to affect this biological process.

In summary, we provide evidence of a novel positive feedback loop involving canonical Wnt signaling and miR218 and likely contributing to human myocardial proliferation and differentiation. This novel regulatory circuit provides additional insight into how miRNAs interact with signaling molecules during myocardial proliferation and differentiation.

## Materials and Methods

### Isolation and identification of CSCs

CSCs were isolated from the hearts of Balb/c mice (18–25 g) using a previously published method^38^, with one minor modification. All of the Balb/c mice were obtained from the Laboratory Animal Science Department of the Second Affiliated Hospital of Harbin Medical University, Heilongjiang, P.R. China. All experimental animal procedures were approved by the Local Ethics Committee for Animal Care and Use at Harbin Medical University in accordance with the guidelines of Directive 2010/63/EU of the European Parliament on the protection of animals used for scientific purposes and NIH guidelines. Briefly, the mice were injected with heparin (5,000 IU/kg, i.p.) 20 min prior to the initiation of the experimental protocol and were subsequently sacrificed through cervical dislocation. The heart was excised, and the aorta was rapidly cannulated. The cannulated heart was mounted on a Langendorff perfusion apparatus with constant flow, and the perfusion pressure was monitored. The heart was initially perfused with Ca_2_^+^ -free Tyrode solution for 10 min to remove blood and was subsequently digested using 0.5 mg/ml collagenase (Sigma, St-Louis, MO, USA) and 0.5 mg/ml trypsin (GIBCO, Invitrogen Inc., Carlsbad, CA, USA) at 37 °C for 30 min. The heart tissue was subsequently minced, and the cell suspension was filtered with a Steriflip (SCNY00100-1EA, Millipore corp., Billerica, MA, USA). The cells were then incubated with a FITC rat anti-mouse CD117/c-kit antibody (BD Biosciences, Franklin Lake, NJ, USA) and separated using MACS anti-FITC microbeads (Miltenyi Biotec, Bergisch Gladbach, Germany). Small round cells, containing most of the c-kit + population, were obtained and cultured for 3–5 days in HyClone Dulbecco’s MEM/F12 (Thermo Fisher Scientific, Waltham, MA, USA) containing fetal bovine serum (FBS), 10 ng/ml bFGF (PeproTech, Rocky Hill, NJ, USA), 10 ng/ml IGF (PeproTech), 10 ng/ml EGF (PeproTech) and 10 ng/ml LIF (Sigma) at 37 °C. After recovery, the cells were used for experiments. We have previously shown that CSCs maintain phenotypic features of stemness and display some characteristics of the early cardiac phenotype. All animal experiments were approved by the Animal Ethics Committee (CEEA).

### Cell differentiation

CSCs at passage 3–5 were cultured at a density of 2 × 10^4^ cells/cm^2^ in differentiation medium containing DMEM, 2% FBS, 100 nM oxytocin acetate, 50 μg/mL ascorbic acid, 10 M 5-azacytidine and 10 mM beta-glycerol phosphate. The medium was exchanged every 3–4 days until the indicated days.

### Treatments

**miRNA transfection.** For miR218 upregulation or knockdown, an miR218 mimic and inhibitor were synthesized as unconjugated and fully phosphorothioated mixed DNA oligonuleotides with a 6-carboxyfluorescein (FAM) moiety at the 5′ end (Shanghai GenePharma Co., Ltd, Shanghai, China). Cells were transfected with 2 μg of the mimic or inhibitor in six-well plates using a commercial transfection reagent (X-tremeGene siRNA Transfection reagent, Roche Applied Science, Penzberg, Germany), according to the manufacturer’s instructions.

### Wnt1 gene transfection

We have previously shown that the transfection efficiency of pEGFP-C3-Wnt1 fusion plasmids is 32.2 ± 0.26% (p < 0.05) in CSCs. In the present study, we arrested the lenti-shRNA targeting packaging plasmids and co-transfected the plasmids into 293FT packaging cells. After 48 h of incubation, we collected the lentivirus released into the conditioned medium, filtered the medium, and used the supernatant to infect CSCs. After 2 weeks of antibiotic selection, stable clones were obtained and were subsequently confirmed through PCR and DNA sequencing analysis.

### ShRNA-sFRP2 transfection

The effective RNAi sequence targeting the sFRP2 gene was designed and screened at the Shanghai Bioladder Corporation (Shanghai, China). We initially selected three sequences as target sequences for sFRP2: (1) sFRP2 shRNA1 5′-GAT CCG CGC AGC AAT TGC AAG CCC TTC AAG AGA GGG CTT GCA ATT GCT GCG CTT TTT TGG AAA-3′; (2) sFRP2 shRNA2 5′- GAT CCG CGA CAT AAT GGA AAC GCT TTT CAA GAG AAA GCG TTT CCA TTA TGT CGT TTT TTG GAA A-3′; and 3) sFRP2 shRNA3 5′-GAT CCG TTC CTG TGC TCG CTC TTC TTC AAG AGA GAA GAG CGA GCA CAG GAA CTT TTT TGG AAA-3′; Control shRNA sequence: 5′-GAT CCA CTA CCG TTG TTA TAG GTG TTC AAG AGA CAC CTA TAA CAA CGG TAG TTT TTT GGA AA-3′. The cDNA containing both the sense and antisense oligonucleotides of the targeting sequences was designed, synthesized, and cloned into the Plk0.1-GFP-SP6 vector to construct a lentiviral vector expressing sFRP2 shRNA. We arrested the lenti-shRNA targeting packaging plasmids and co-transfected the plasmids into 293FT packaging cells. After 48 h of incubation, we collected the lentivirus released into the conditioned medium, filtered the medium, and used the supernatant to infect CSCs. After 2 weeks of antibiotic selection, stable clones were obtained and were subsequently confirmed through PCR and DNA sequencing analysis. After assessment, shRNA2 was selected as the most efficient sequence for blocking sFRP2 expression.

### siRNA-β-catenin transfection

Gene silencing by small interfering RNA (siRNA) involves a small double-stranded RNA that degrades target mRNA. A β-catenin-siRNA duplex was synthesized at Shanghai GenePharma Co., Ltd (Shanghai, China) (sense: 5′-GCC UCU GAU AAA GGC AAC UTT-3′; antisense: 5′-AGU UGC CUU UAU CAG AGG CTT-3′). In the control group, the cells were treated with either transfection reagents (vehicle) or a non-targeting siRNA (siRNA-NC) (sense: 5′-UUC UCC GAA CGU GUC ACG-3′; antisense: 5′-ACG UGA CAC GUU CGG AGA ATT-3′). The cells were transfected using the X-treme siRNA Transfection Reagent (Roche Applied Science, Penzberg, Germany), according to the manufacturer’s instructions.

### Microarray analysis of miR expression

Low-molecular-weight RNA was isolated from proliferating and differentiated CSCs using the mirVana™ RNA Isolation Kit (Ambion). The miRNA expression profile was determined through microarray analysis using the μParaflo™ microfluidic chip (MiHuman_8.2- Based on Sanger miRBase Release 8.2, LC Sciences), according to the manufacturer’s instructions.

### Construction of luciferase reporter gene plasmids

Four types of oligonucleotides were used, referred to as the wild-type, mutation-1, mutation-2 and double mutation oligonucleotides. All oligonucleotides were annealed with the following parameters: 95 °C for 5 min and 85 °C for 5 min, followed by 75 °C for 5 min and 70 °C for 5 min. The Psicheck-2 vector was cut by *Xho*I, and the fragment was isolated using the QIAquick Gel Extraction Kit (Qiagen, Venlo, Netherlands). The oligonucleotides and the fragments from the Psicheck-2 vector were ligated using T4 DNA ligase. Five clones from each type of oligonucleotide were cultured, and the plasmids were obtained. The successful constructs were confirmed through restriction enzyme digestion and sequencing.

### Luciferase activity assay

Promoter activity was evaluated using a Dual Luciferase Reporter Assay Kit (Promega, Madison, WI, USA), according to the manufacturer’s instructions. The transfected cells were washed once with phosphate-buffered saline and lysed in passive lysis buffer for 15 min with gentle agitation. Next, 100 μl of LAR II was added to labeled luminometer tubes to complete the DLR™ assays. The luminometer was set to perform a 2-sec premeasurement delay, followed by a 10-sec measurement period for each reporter assay. A 20-μl sample of the cell lysate was carefully transferred to the luminometer tube containing LAR II, and the solution was mixed after pipetting 2–3 times. The tubes were placed in the luminometer, and the intensity was measured. Subsequently, the sample tubes were removed from the luminometer, and 100 μl of Stop & Glo^®^ Reagent was added. After brief vortexing, the samples were again measured in the luminometer.

### Flow cytometry

A single cell suspension of 0.5–1.0 × 10^6^ cells/mL in phosphate-buffered saline was incubated at a 1:100 dilution at 4 °C for 30 min in the dark for tagging with the following fluorescent primary antibodies: CD29-FITC, CD90-PE, c-kit(CD117)-FITC, sca-1-FITC, and CD34-PE (BD Biosciences, Franklin Lake, NJ, USA). A total of 10,000 events were acquired using a BD LSRII flow cytometer (BD Biosciences), and the data were analyzed using BD FACSDiva^TM^ Software. Flow cytometry was performed in duplicate using cells from three independent experiments.

### Immunofluorescence

To characterize cell differentiation among isolated cells, the cells were fixed with 4% (v/v) formaldehyde for 15 min. After washing with PBS, the cells were blocked with 10% BSA and incubated at 37 °C for 1 h with mouse anti-cardiac myosin heavy chain antibodies (1:100, ab15, Abcam Ltd., Cambridge, MA, USA) and mouse anti-Tn-I (1:100,ab19615, Abcam Ltd.). The cells were subsequently washed and incubated in the dark for 1 h at 37 °C with Rhodamine (TRITC)-conjugated AffiniPure Goat Anti-Mouse IgG (H+L)-antibodies (1:200, ZSGBBIO, Beijing, China). After washing, the nuclei were counterstained with 49,6-diamidino-2-phenylindole dihydrochloride (DAPI, Sigma, St Louis, MO, USA). The cells were subsequently examined under a fluorescence microscope (DMI4000B, Leica, Germany).

### Cell proliferation assay

The proliferation of CSCs was determined through 5-ethynyl-2-deoxyuridine (EdU) incorporation (RiboBio, Guangzhou, China). The cells were fixed and stained after incubation according to the manufacturer’s instructions. The proliferation rate was calculated after normalizing the number of EdU-positive cells to the number of Hoechst 33342-stained cells in five random fields.

For cell cycle analysis, the cells were harvested at 48 h after transfection with the miR218 mimic or inhibitor, then washed twice with PBS and fixed in 75% ethanol overnight. The next day, the cells were washed twice with PBS and incubated in RNaseA (20 mg/ml) at 37 °C for 30 min, followed by staining with propidium iodide (PI, 0.5 mg/ml) at 4 °C for 30 min. The cells were subsequently washed and resuspended in 500 mL of PBS, followed by the detection of the DNA contents using a Becton-Dickinson flow cytometer (BD Diagnostics, Sparks, MD, USA).

### Quantitative RT-PCR

Total RNA was isolated with TRIzol (Invitrogen Inc., Carlsbad, CA, USA) from the cells subjected to the different experimental conditions using according to the manufacturer’s instructions. After pretreatment using RNase-free DNase I, 2 μg of total miRNA was subjected to the protocols of the miScript Reverse Transcription Kit and the miScript SYBR Green PCR kit (Qiagen, Venlo, the Netherlands), and the Transcriptor First Strand cDNA Synthesis Kit and FastStart Universal SYBR Green Master Mix (ROX) were used for reverse transcription. Gene amplification was confirmed after calculating the melting temperatures (Tm) for the products from the melting peak curve (2^dF/dT^ vs. temperature).

All amplicons were collected and confirmed through agarose gel electrophoresis and sequencing. A cross-point vs. logarithmic concentration standard curve was generated using serial dilutions of one of the cDNA samples or known concentrations of plasmid DNA with a Wnt1 gene insert. Negative controls were included, using cDNAs synthesized in the same manner as described above, but without reverse transcriptase. Each cDNA sample was run in triplicate. The data were averaged, and standard deviations were calculated. The GAPDH gene was used as a standard control. U6 was employed for miR218 template normalization. The primers used in the present study are described in Table 1.

### TOPflash reporter assay

The wild-type TOPflash β-catenin LUC reporter (200 ng) (Addgene, Inc., Cambridge MA) was co-transfected with the mimic-control, miR-218 mimic, inhibitor-control and miR218 inhibitor, along with 5 ng of a Renilla LUC reporter plasmid using Oligofectamine (Invitrogen) in CSCs (60% confluency). Transfection was performed according to the manufacturer’s protocol. The transfected cells were incubated at 37 °C for 24 h and assayed for relative luciferase activity normalized to Renilla values.

### Western blotting

Protein samples were denatured at 95 °C for 5 min before being loaded onto an SDS-polyacrylamide gel (reducing or nonreducing, 4–15%) and run under standard conditions. The proteins were then transferred to polyvinylidene difluoride membranes (PVDF, Millipore corp., Billerica, MA, USA). The membranes were blocked with 5% non-fat milk at room temperature for 1 h in Tris-buffered saline containing Tween 20 (TBST). Primary antibodies against p-GSK-3β and β-catenin (Cell Signaling, Danvers, MA, USA), troponin I (ab19615, Abcam, Cambridge, MA, USA), the α-myosin heavy chain (ab15, Abcam Ltd.) or β-actin (Zhongshan Goldenbridge Biotechnology Co. Ltd., Beijing, China) were incubated overnight with the membranes at 4 °C. The membranes were subsequently incubated for 60 min with peroxidase-conjugated Affinipure goat anti-rabbit IgG (H+L) and anti-mouse IgG (H+L)-labeled secondary antibodies diluted 1:2000. The membranes were washed in TBST with 0.5% Tween-20 before ECL detection with BeyoECL Plus (Beyotime Institute of Biotechnology, Haimen, China). After exposure of an X-ray film, the blot was stripped in 5 ml of stripping buffer (CoWin Biotech, Beijing, China) for 15 min at room temperature and hybridized with an antibody against β-actin for normalization. Densitometric analysis of the resultant protein bands was performed using the Tanon Gel Imaging System (Shanghai Tanon Co. Ltd., Shanghai, China).

### Statistical analysis

Statistical analysis was performed using SPSS 18.0 (SPSS Inc., Chicago, IL, USA). The measurements are presented as the means ± SD. Comparisons of all pairs were performed using Student’s t-test or the least significant difference (LSD) test, as appropriate. P-values of < 0.05 were considered significant. The correlation between miR218 expression and the protein levels of the target genes was examined through Pearson’s correlation analysis. The results are shown as the means ± SD of at least three separate experiments. P < 0.01.

## Additional Information

**How to cite this article**: Wang, Y. *et al*. MiR218 Modulates Wnt Signaling in Mouse Cardiac Stem Cells by Promoting Proliferation and Inhibiting Differentiation through a Positive Feedback Loop. *Sci. Rep.* **6**, 20968; doi: 10.1038/srep20968 (2016).

## Figures and Tables

**Table 1:** Primers used for RT-PCR assays.

Primer	Sequence (5′-3′)
miR218	F: GGA AAT CCC TGG CAA TGT GAT
miR218-1	F: CCA TGG AAC GTC ACG CAG C
miR218-2	F: GCG GAA AGC ACC GTG CTC
sFRP2	F: CGT GGG CTC TTC CTC TTG
	R: ATG TTC TGG TAC TCG ATC CG
Tcf-1	F: AGC TTT CTC CAC TCT ACG AAC A
	R: AAT CCA GAG AGA TCG GGG GTC
LEF-1	F: TGT TTA TCC CAT CAC GGG TGG
	R: CAT GGA AGT GTC GCC TGA CAG
Cyclin D1	F: GCG TAC CCT GAC ACC AAT CTC
	R: ACT TGA AGT AAG ATA CGG AGG GC
β-catenin	F: CTG CTC ATC CCA CTA ATG TC
	R: CTT TAT TAA CTA CCA CCT GGT CCT
Cardiac-α-actin	F: CTG AGA TGT CTC TCT CTC TCT TAG
	R: ACA ATG ACT GAT GAG AGA TG
α-myosin heavy chain	F: GGA AGA GTG AGC GGC CAT CAA GG
	R: CTG CTG GAG AGG TTA TTC CTC G
Wnt1	F: GGA AGA GTG AGC GGC CAT CAA GG
	R: CTG CTG GAG AGG TTA TTC CTC G
GAPDH	F: AGG TCG GTG TGA ACG GAT TTG
	R: TGT AGA CCA TGT AGT TGA GGT C
U6	F: CGC TTC GGC AGC ACA TAT AC
	R: AAA ATA TGG AAC GCT TCA CGA
