# Author Correction: MiR218 Modulates Wnt Signaling in Mouse Cardiac Stem Cells by Promoting Proliferation and Inhibiting Differentiation through a Positive Feedback Loop

**DOI:** 10.1038/s41598-021-91687-1

**Published:** 2021-06-03

**Authors:** Yongshun Wang, Jingjin Liu, Jinjin Cui, Meng Sun, Wenjuan Du, Tao Chen, Xing Ming, Lulu Zhang, Jiangtian Tian, Ji Li, Li Yin, Fang Liu, Zhongyue Pu, Bo Lv, Jingbo Hou, Bo Yu

**Affiliations:** 1 Cardiology Department, Second Affiliated Hospital of Harbin Medical University, Harbin, Heilongjiang Province China; 2 Key Laboratories of the Education Ministry for Myocardial Ischemia Mechanisms and Treatment, Harbin, Heilongjiang Province China

Correction to: *Scientific Reports* 10.1038/srep20968, published online 10 February 2016

This Article contains errors in Figure 4 and 6.

In the assembly of Figure 4B, images from Figure 2C were inadvertently incorporated for the “shRNA-sfrp2”, “shRNA-sfrp2 + miR218”, and “miR218 inhibitor” panels.

For Figure 4C, the flow cytometry data for the “shRNA-sfrp2 + miRNA217 inhibitor” and “miR218 inhibitor” panels were incorrect.

In Figure 6A, incorrect images were used for the “mimic con” condition.

The correct Figures 4 and 6 and accompanying legends appear below. All data in Figure 4C has been replaced, to ensure the presented flow cytometry images exhibit the same gating.

**Figure 4:**
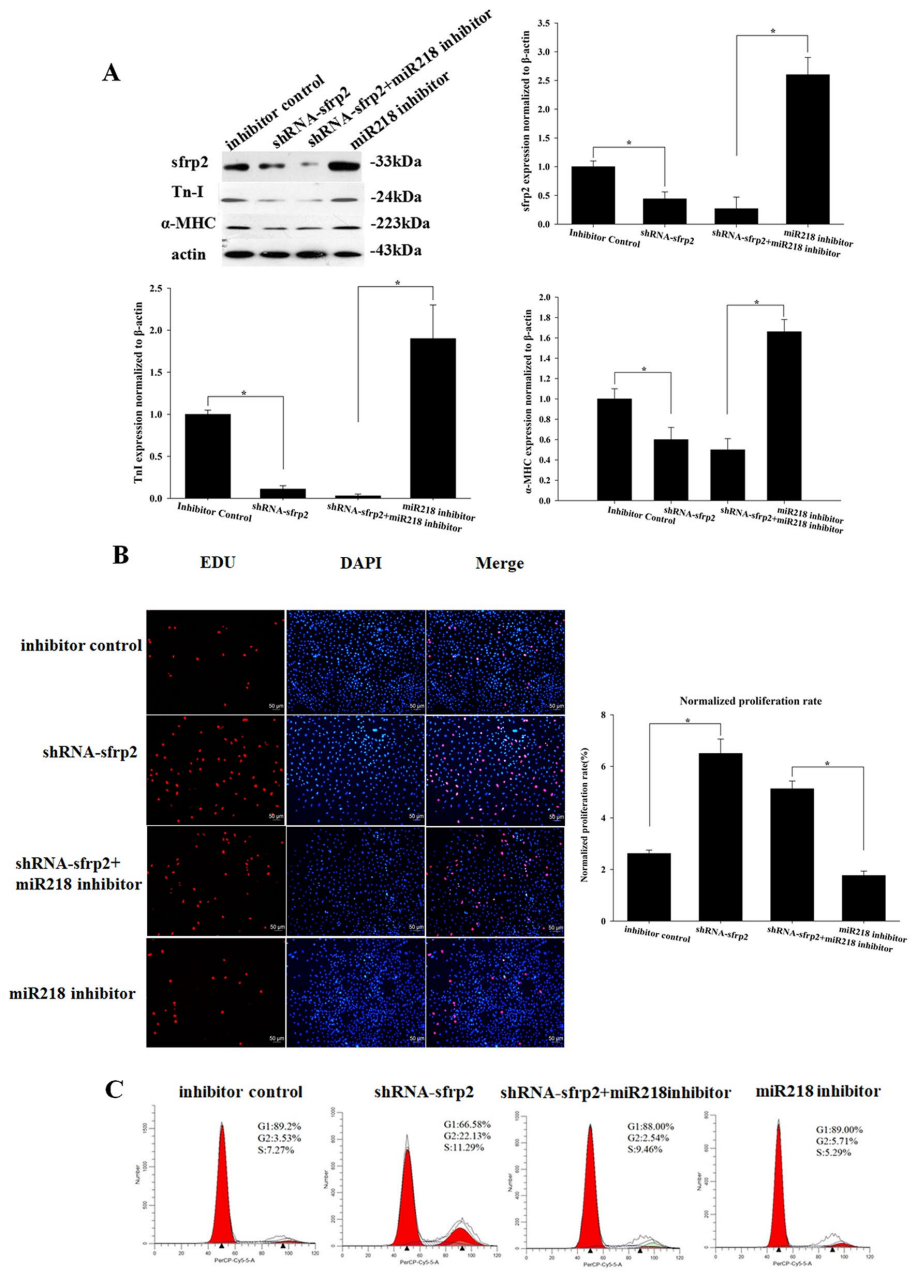
MiR218 regulates cell proliferation and differentiation by targeting sFRP2. (**A**) Western blot analysis of proteins from CSCs transfected with sFRP2 shRNA, the miR218 inhibitor + sFRP2 shRNA and the miR218 inhibitor and subsequently cultured in differentiation media for 12 days. The protein profiles were normalized to β-actin. (**B**) The cells were stained with EdU and Hoechst 33342. (**C**) Cell cycle distribution of CSCs after transfection with sFRP2 shRNA, the miR218 inhibitor + sFRP2 shRNA and the miR218 inhibitor for 48h.

**Figure 6:**
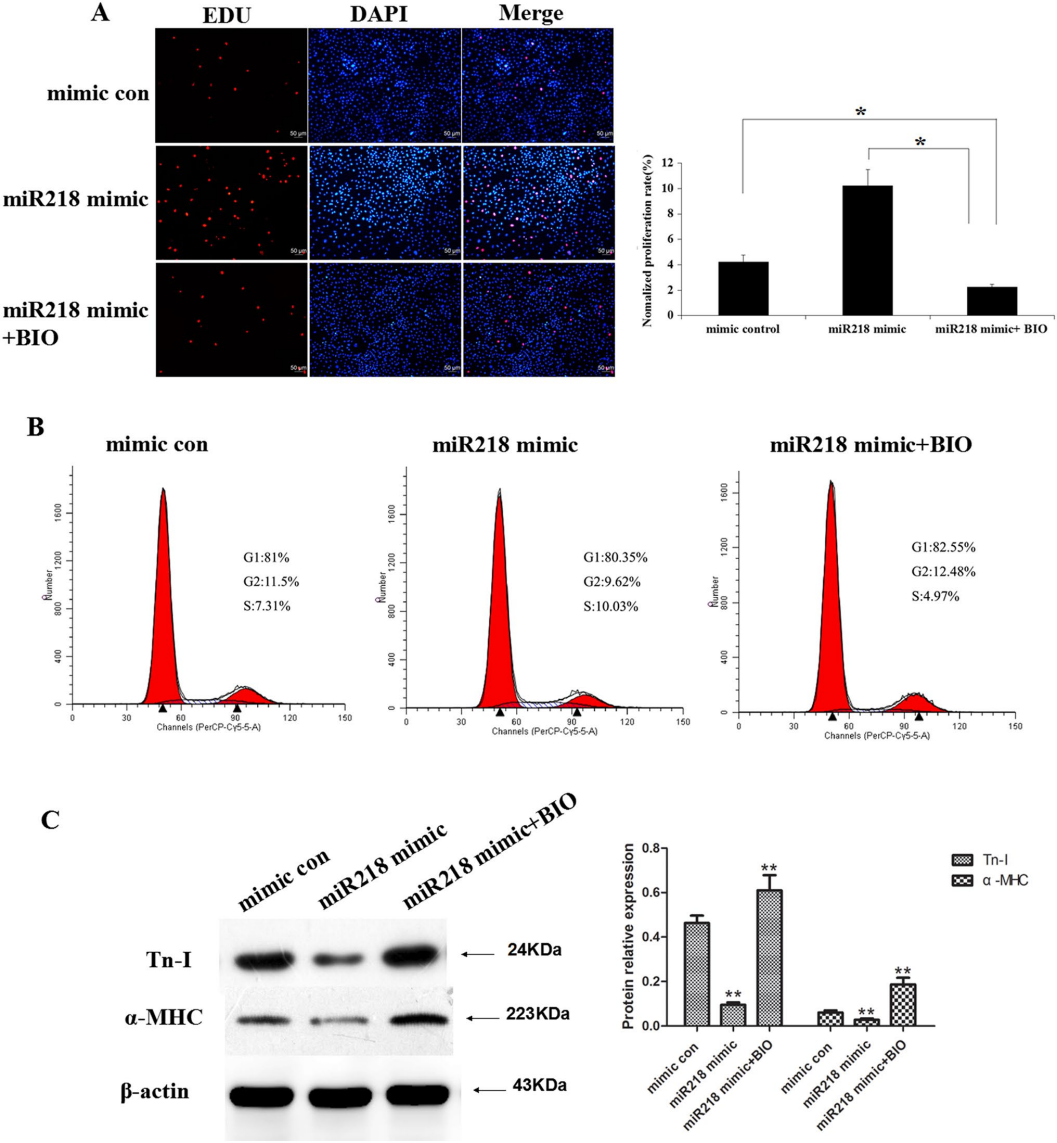
BIO restored the effects of miR218 on the proliferation and cardiac differentiation of CSCs. (**A**) Cells transfected with the miR218 mimic and treated with BIO were stained with EdU and Hoechst 33342. (**B**) Cell cycle distribution of the CSCs after the same treatment. (**C**) Western blot analysis of proteins from CSCs transfected with the mimic-control or the miR218 mimic or treated with the miR218 mimic and BIO and subsequently cultured in differentiation medium for 12 days.

